# Cholinergic stimulation improves electrophysiological rate adaptation during pressure overload-induced heart failure in rats

**DOI:** 10.1152/ajpheart.00293.2020

**Published:** 2020-10-02

**Authors:** Frederick M. Zasadny, Jhansi Dyavanapalli, N. Maritza Dowling, David Mendelowitz, Matthew W. Kay

**Affiliations:** ^1^Department of Biomedical Engineering, School of Engineering and Applied Science, The George Washington University, Washington, District of Columbia; ^2^Department of Pharmacology and Physiology, School of Medicine and Health Sciences, The George Washington University, Washington, District of Columbia; ^3^Department of Acute and Chronic Care, School of Nursing, The George Washington University, Washington, District of Columbia; ^4^Department of Epidemiology and Biostatistics, Milken School of Public Health, The George Washington University, Washington, District of Columbia

**Keywords:** electrophysiology, heart failure, neurocardiology, optical mapping, parasympathetic stimulation

## Abstract

Left ventricular (LV) electrical maladaptation to increased heart rate in failing myocardium contributes to morbidity and mortality. Recently, cardiac cholinergic neuron activation reduced loss of contractile function resulting from chronic transverse-ascending aortic constriction (TAC) in rats. We hypothesized that chronic activation of cardiac cholinergic neurons would also reduce TAC-induced derangement of cardiac electrical activity. We investigated electrophysiological rate adaptation in TAC rat hearts with and without daily chemogenetic activation of hypothalamic oxytocin neurons for downstream cardiac cholinergic neuron stimulation. Sprague–Dawley rat hearts were excised, perfused, and optically mapped under dynamic pacing after 16 wk of TAC with or without 12 wk of daily chemogenetic treatment. Action potential duration at 60% repolarization (APD_60_) and conduction velocity (CV) maps were analyzed for regional rate adaptation to dynamic pacing. At lower pacing rates, untreated TAC induced elevated LV epicardial APD_60_. Fitted APD_60_ steady state (APD_ss_) was reduced in treated TAC hearts. At higher pacing rates, treatment heterogeneously reduced APD_60_, compared with untreated TAC hearts. Variance of conduction loss was reduced in treated hearts compared with untreated hearts during fast pacing. However, CV was markedly reduced in both treated and untreated TAC hearts throughout dynamic pacing. At 150 ms pacing cycle length, APD_60_ versus diastolic interval dispersion was reduced in treated hearts compared with untreated hearts. Chronic activation of cardiac cholinergic neurons improved electrophysiological adaptation to increases in pacing rate during the development of TAC-induced heart failure. This provides insight into the electrophysiological benefits of cholinergic stimulation as a treatment for patients with heart failure.

**NEW & NOTEWORTHY** Analysis of electrophysiology from optical mapping of failing left ventricular myocardium provided insight into the possible therapeutic outcomes of cholinergic stimulation within the left ventricle. Chronic hypothalamic oxytocin neuron activation for downstream cardiac cholinergic neuron stimulation blunted onset of failing electrophysiology induced by pressure overload-induced heart failure in rats.

## INTRODUCTION

Despite major medical advances, morbidity and mortality from cardiac arrhythmia remain high in patients with heart failure (5, 8, 22, 35). Within failing myocardium, action potential (AP) prolongation (46), alterations in conduction (26), altered sodium and calcium homeostasis (6), autonomic imbalance (41), and maladaptive signaling create an arrhythmogenic substrate (47). Both reentrant and focal mechanisms have been reported in the initiation and maintenance of ventricular tachyarrhythmias within failing myocardium (12, 21, 26, 39). In either mechanism, the amount by which the electrical activity of cardiomyocytes adapts to changes in heart rate, quantified by restitution curves, influences arrhythmia maintenance (16, 50). This adaptation is observed as reductions in effective refractory period and action potential duration (APD) as well as slowing of myocardial conduction velocity (CV) with increased heart rate (9, 11). Within failing myocardium, electrophysiological rate adaptation remains incompletely understood.

We examined the hypothesis that during pressure overload-induced heart failure, epicardial depolarization and repolarization would fail to adapt to increases in heart rate. We studied a rat model of early-onset heart failure from transverse-ascending aortic constriction (TAC) by optically mapping left ventricular (LV) epicardial electrophysiology. Epicardial APD, CV, conduction failure, and APD versus DI dispersion were examined during dynamic pacing to assess electrophysiological responses to chronotropic stress. These parameters were significantly altered after 16 wk of TAC.

As effective heart failure treatment remains elusive (34), increasing cardiac parasympathetic tone has been shown to blunt maladaptive ventricular remodeling (4, 42) and decrease mortality and morbidity in models of heart failure (14, 19, 31). Consequently, we examined the hypothesis that increased stimulation of the cardiac parasympathetic network during the development of TAC-induced heart failure would blunt alterations in ventricular electrophysiology. We did this by daily activation of excitatory designer receptors exclusively activated by designer drugs (DREADDs) expressed in oxytocin neurons of the paraventricular nucleus (PVN) (14, 17, 23). These neurons are known to monosynaptically synapse upon and excite premotor cardiac vagal neurons in the brainstem (38), which in turn are well known to activate postganglionic parasympathetic cholinergic neurons that release acetylcholine within the myocardium. In this study, such chronic cholinergic stimulation blunted TAC-induced electrophysiological changes by preserving regional conduction continuity and repolarization rate response at high pacing rates.

## METHODS

### 

#### Animals.

Male Sprague-Dawley rats were bred and housed at the George Washington University’s Animal Research Facility. Rats were fed standard chow ad libitum with free access to water in single housing with a 12-h:12-h light-dark cycle. Rats underwent additional in vivo assessments for parallel research studies. All animal procedures were approved by the George Washington University’s Institutional Animal Care and Use Committee.

#### Transverse-ascending aortic constriction.

TAC surgery was performed at 1 wk of age, as previously reported (14, 17). Briefly, a 4-0 suture was placed on the ascending aorta and tightened around a 25-gauge needle. Upon removal of the needle, the aorta was constricted to a uniform diameter. In sham surgeries, the suture was not tied. The effect of the constriction was fully manifest near 3 wk of age due to aortic growth into the suture. After TAC surgery, rats were housed for 16 wk, then euthanized for ex vivo optical mapping experiments. The suture and aortic constriction were visualized upon excision of the heart.

#### PVN oxytocin neuron activation.

Hypothalamic PVN oxytocin neurons were targeted to selectively express DREADDs as previously reported (14, 17, 23). At 1 wk of age, a combination of adeno-associated viruses (AAVs) was injected into the PVN. The injection contained an AAV-expressing Cre recombinase under an oxytocin promoter and an AAV-expressing hM_3_Dq DREADDs in a Cre-dependent manner. This combination allowed selective expression of DREADDs within PVN oxytocin neurons as previously reported (23). To stimulate these neurons, daily intraperitoneal injections of clozapine-*N*-oxide (CNO) at 1 mg/kg body wt (or saline for untreated rats) began at 5 wk of age and continued until euthanasia at 16 wk of TAC. CNO is variably converted to clozapine, which passes the blood-brain barrier and binds to DREADDs (18, 33). Off-target effects of converted clozapine were not studied.

#### Groups.

The following three groups were analyzed: Sham (*n* = 7), TAC (*n* = 5), and TAC + CNO (*n* = 7). Upon euthanasia, DREADDs expression within PVN oxytocin neurons was confirmed by microscopy of a coexpressed fluorescent tag. Most saline-injected rats had the DREADDs injection, and all were included in analysis even if labeling was not detected. However, CNO-injected rats were excluded if no labeling was detected, with exception of inconclusive negative labeling in one TAC + CNO rat.

#### Langendorff perfusion.

At 16 wk of TAC, rats were placed in a deep surgical plane of anesthesia by isoflurane inhalation, confirmed by lack of pedal reflex, and extremely reduced breath rate. The heart was then quickly excised via thoracotomy and placed in cold media to slow heart rate and aid aortic cannulation. The heart was then Langendorff perfused at constant aortic pressure (75 mmHg) with 37°C perfusate oxygenated with 95% O_2_-5% CO_2_. Perfusate contained (in mM) 118 NaCl, 4.7 KCl, 1.25 CaCl_2_, 0.57 MgSO_4_, 1.17 KH_2_PO_4_, 25 NaHCO_3_, and 6 mM glucose. The heart was rotated for anterior LV epicardial optical mapping while partially submerged in recirculating perfusate. Electrocardiogram electrodes and a thermocouple monitored heart rate and bath temperature.

#### Optical mapping.

**Fig. 1.**
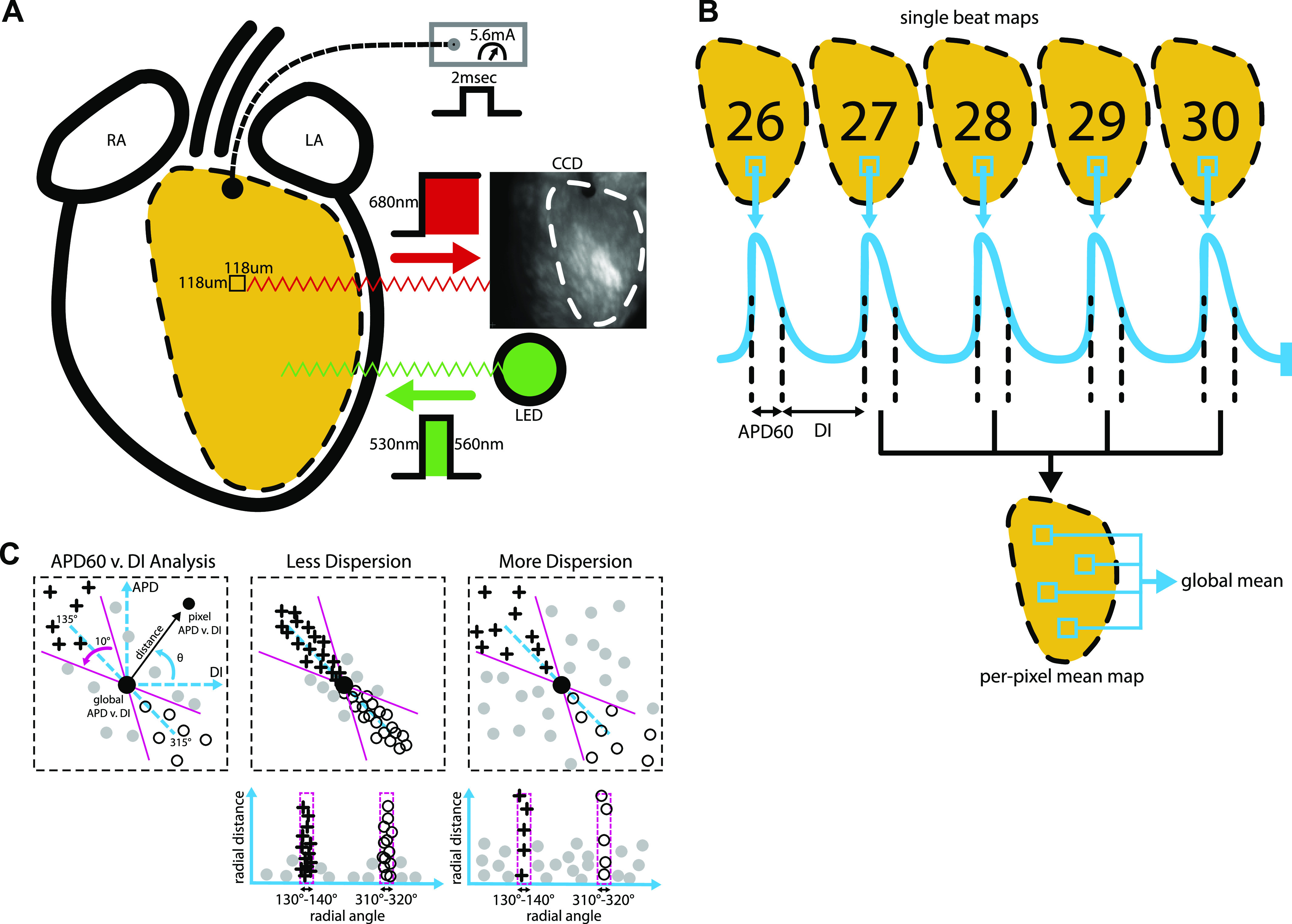
Optical mapping and signal analysis. *A*: schematic of RH237 fluorescence image acquisition showing excitation and emission wavelengths, the location of the pacing electrode, and LV epicardial region that was mapped. *B*: illustration of beat analysis for the last five beats of each PCL within the dynamic pacing protocol. After signal measurement for each pixel, per-pixel means were computed from the last four beats. Global means were then computed from per-pixel means. *C*: APD_60_ vs. DI dispersion analysis diagrams. *Left*: scatterplot showing APD_60_ vs. DI at one PCL and the predicted relationship PCL = APD + DI (blue dotted line). APD_60_ vs. DI radial distance (black arrow) and radial angle (θ) were measured to quantify the location of each pixel’s APD_60_ vs. DI point from the global mean APD_60_ vs. DI restitution point (large filled circle). *Middle*: scatterplot showing low APD_60_ vs. DI dispersion (*top*), with most points falling along the predicted line. Radial distance vs. radial angle plots of low APD_60_ vs. DI dispersion (*bottom*) have most points falling within 10° segments centered at 135° (crosses) and 315° (open circles). *Right*: scatterplot showing high APD_60_ vs. DI dispersion (*top*), with many points falling off the predicted line. Radial distance vs. radial angle plots of high APD_60_ vs. DI dispersion (*bottom*) have many points falling between 135° and 315°. APD_60_, action potential duration at 60% repolarization; DI, diastolic interval; LV, left ventricular; PCL, pacing cycle length.

After a brief period of equilibration, hearts were excitation-contraction uncoupled with a slow bolus injection of blebbistatin (Sigma-Aldrich) into aortic flow for a final circulating concentration of 7 μM. After contractile arrest, a bolus of the fast-response potentiometric fluorescent probe RH237 (Invitrogen) with 528 nm/782 nm excitation/emission was injected into aortic flow for a final concentration of 0.7 μM. RH237 was excited using two 530-nm light-emitting diodes (Mightex), each with an added 530–560 nm bandpass filter (Chroma). Excitation light intensity varied between several hearts due to photobleaching concerns. Shifts in RH237 emission peak due to changes in epicardial cardiomyocyte membrane potential were acquired with a high speed 128 × 128 pixel (118 μm spatial resolution) EMCCD camera (Andor iXon DV860) with an added 680-nm long-pass filter (Chroma) (Fig. 1*A*).

Hearts were dynamically paced (2 ms pulse duration, 5.6 mA amplitude) during optical mapping using an electrode placed on the base of the LV epicardium (Fig. 1*A*). The dynamic pacing protocol consisted of 30 pulses for each consecutive pacing cycle length (PCL) of 250, 200, 175, 150, 125, 97, 80, 70, and 60 ms. Frames were recorded throughout pacing at 490.2 frames/s.

#### Image processing.

Optically mapped fluorescence was analyzed using in-house MATLAB software to measure the time of depolarization and repolarization at each pixel. A region of interest for analysis was drawn to include the LV, but to exclude the right ventricle (RV), atria, and curved edges of the LV. Residual motion artifact was removed through nonrigid registration of the raw fluorescence data, as described by Christoph and Luther (13). Saturated pixels and pixels with low fluorescence signal variance were excluded. Signals at each pixel were corrected for baseline drift by subtracting the linear trend and temporally filtered using a low-pass 40–45 Hz Butterworth filter (2). Background fluorescence was then subtracted, pixel signals were normalized from 1 to 100, and the images were spatially averaged using a 5 × 5 mean filter.

#### Optical AP analysis.

Intervals of mapping data corresponding to the last five optical APs of PCLs 250–97 ms were selected for analysis (Fig. 1*B*). PCLs were not analyzed when pacing capture was lost. Activation time (AT) at each pixel was calculated as the time of 50% of optical AP upstroke amplitude. AP upstroke amplitude was calculated from the maximal inflection point at the base of the upstroke to the AP peak. ATs above and below 2.5 standard deviations from the mean of all ATs for a paced beat were removed from analysis. Action potential duration at 60% repolarization (APD_60_) was measured to assess ion channel activity in the slower repolarization phase of the AP, an interval that has been shown to be altered during volume overload-induced heart failure in rats (44). APD_60_ was computed as the difference between the time at 40% of optical AP upstroke amplitude in the repolarization phase and the AT (Fig. 1*B*). Diastolic intervals (DIs) were then computed by subtracting APD_60_ from the interval between the AT and the next AT. Notably, this DI definition includes the remaining 40% of AP repolarization. CV was calculated by fitting a plane to a local spatial distribution (*x,y,t*) of ATs (3). Fits were constrained to a spatial window centered at each pixel and a time window that varied with the spatial gradient of AT.

APD_60_ and CV were then averaged at each pixel using the last four consecutive optical APs (beats 27, 28, 29, and 30) of each PCL (Fig. 1*B*). DIs were also averaged at each pixel for beats 26, 27, 28, and 29 of each PCL, providing an average measure of previous DI to be matched with average APD_60_ and CV. If an activation or repolarization time was not detected for an AP at a pixel, then that AP was not included in the per-pixel mean or any other analysis. Per-pixel APD_60_ mean maps were generated using the average at each pixel. Histograms of per-pixel APD_60_ and CV mean values were created with fixed centers. Conduction loss was calculated as the percent change from 250 ms PCL to 150 ms PCL in the number of pixels that included four APs within the per-pixel mean. The number of APs used for each per-pixel mean was also mapped to show regional conduction loss.

#### Restitution curve analysis.

A PCL’s global mean was calculated using the APD_60_, CV, and DI per-pixel mean values. Restitution plots were constructed by plotting the global mean APD_60_ (or CV) versus DI per PCL for each heart. APD_60_ restitution vector angle was calculated from the positive horizontal (right hand) to the vector pointing from the 175 ms PCL restitution point to the 150 ms PCL restitution point. Similarly using per-pixel APD_60_ and DI mean values for per-pixel restitution points, a vector angle at each pixel was also computed and mapped to show dispersion of restitution vector angle.

Restitution data were fitted to the monoexponential function APD_60_ = APD_ss_·[1 − *b*·exp(−DI/τ)] (Eq. *1*) using a nonlinear least squares approach in MATLAB. This provided further insight into differences in APD_60_ between the groups in a manner that incorporated the full APD_60_ restitution relationship. Due to the important association between steady-state APD and the APD restitution curve (45), APD_60_ restitution data were fitted to measure steady state at APD_60_ (APD_ss_) for each heart. The parameters of Eq. 1 (APD_ss_, b, and τ) were first fitted using restitution data of all hearts in each group to provide one parameter set for each group. The distribution of APD_ss_ among hearts was then computed for each group by fitting the restitution data of each heart while setting b and τ equal to those of the group fits.

#### APD_60_ vs. DI dispersion analysis.

Per-pixel APD_60_ mean versus per-pixel DI mean was analyzed for dispersion from the global mean restitution point and the predicted PCL = APD + DI line for each PCL (Fig. 1*C*). Radial distance defined the length of a straight line between the global mean restitution point to a per-pixel APD_60_ versus DI point. Radial angle defined the angle of a per-pixel APD_60_ versus DI point from a positive horizontal unit vector originating at the global mean restitution point. The PCL = APD + DI line predicted where a pixel from tissue with appropriate rate adaptation should lie in the APD_60_ versus DI space for a single PCL. A pixel is predicted to lie along this line at 135° or 315° relative to the global restitution mean point. The percent of pixels inside 10° segments centered at 135° and 315° quantified epicardial regions that appropriately adapted to a PCL. Pixels within these 10° segments were marked on per-pixel APD_60_ mean maps.

#### Statistical analysis.

Planned contrast comparisons of Sham versus TAC and TAC versus TAC + CNO were performed after meeting equal variance (Brown–Forsythe) and normality assumptions. Comparisons were conducted using Fisher’s least significant difference test with a single pooled variance in Prism 8 (GraphPad), not correcting for multiple comparisons. A *P* value threshold of less than 0.05 was considered significant. Mean, standard deviation, and test results are displayed in Table 1. Representative hearts (Sham 2063-4, TAC 022-3, and TAC + CNO 3026-2) were selected based on the observed similarity of their per-pixel APD_60_ mean map to their group’s mean maps at 150 ms PCL. Supplementary figures include data for all hearts analyzed (all Supplemental material available at https://doi.org/10.6084/m9.figshare.12200813.v1).

**Table 1.** Optical mapping and anatomic parameters for each group

	Sham		TAC		TAC + CNO				
Parameter		*n*		*n*		*n*	Brown-Forsythe	Sham vs. TAC	TAC vs. TAC + CNO
Optical mapping									
APD_60_, ms									
250 ms PCL	56.41 (8.470)	6	75.78 (10.82)	5	66.56 (12.92)	6	0.7338	0.0108	0.1842
150 ms PCL	55.30 (6.252)	7	58.52 (2.217)	5	53.36 (6.677)	6	0.4341	0.3447	0.1516
APD_ss_, ms	57.90 (6.799)	7	78.27 (7.704)	5	65.46 (10.60)	7	0.5458	0.0010	0.0220
APD_60_ restitution vector angle, °	189.0 (6.943)	7	202.2 (18.93)	5	190.9 (5.661)	6	0.2435	0.0619	0.1176
CV at 250 ms PCL, cm/s	61.00 (12.69)	6	38.95 (4.926)	5	41.78 (5.825)	6	0.1794	0.0010	0.6017
%Change in number of pixels	0.09408 (0.4026)	6	−19.42 (17.08)	5	−0.9820 (1.075)	5	0.0012	N/A	N/A
APD_60_ vs. DI total percentage, %									
250 ms PCL	56.07 (12.88)	6	45.00 (16.78)	5	59.10 (14.00)	6	0.8857	0.2274	0.1303
150 ms PCL	62.66 (12.59)	7	37.28 (12.20)	5	66.46 (11.78)	6	0.8678	0.0029	0.0013
Anatomy									
Body weight, g	513.0 (96.49)	7	382.6 (66.58)	5	444.6 (23.17)	7	0.2121	0.0054	0.1463
LV, mm	3.159 (0.5132)	7	4.350 (0.2904)	5	3.993 (0.5507)	7	0.5213	0.0007	0.2251
Septum, mm	2.334 (0.6166)	7	3.410 (0.3654)	5	3.173 (0.2694)	7	0.3010	0.0009	0.3822
RV, mm	1.670 (0.4181)	7	1.714 (0.2990)	5	1.661 (0.3598)	7	0.9492	0.8414	0.8111

Values are means (SD); *n*, number of samples. Brown-Forsythe test for equal variance *P* values reported. Planned contrast analysis of Sham vs. TAC and TAC vs. TAC + CNO reported with *P* values from Fisher’s least significant difference test. APD_60_, action potential duration at 60% repolarization; APD_ss_, APD at fitted steady state; CV, conduction velocity; DI, diastolic interval; LV, left ventricle; N/A, not applicable; PCL, pacing cycle length; RV, right ventricle. LV, septum, and RV are wall thickness measurements.

## RESULTS

### 

#### Anatomy.

Compared with Sham, rat weight was significantly reduced after 16 wk of TAC (*P* = 0.0054). LV free wall and septum thickness in TAC hearts (*P* = 0.0007 and *P* = 0.0009) were significantly larger than Sham hearts.

#### APD_60_.

**Fig. 2.**
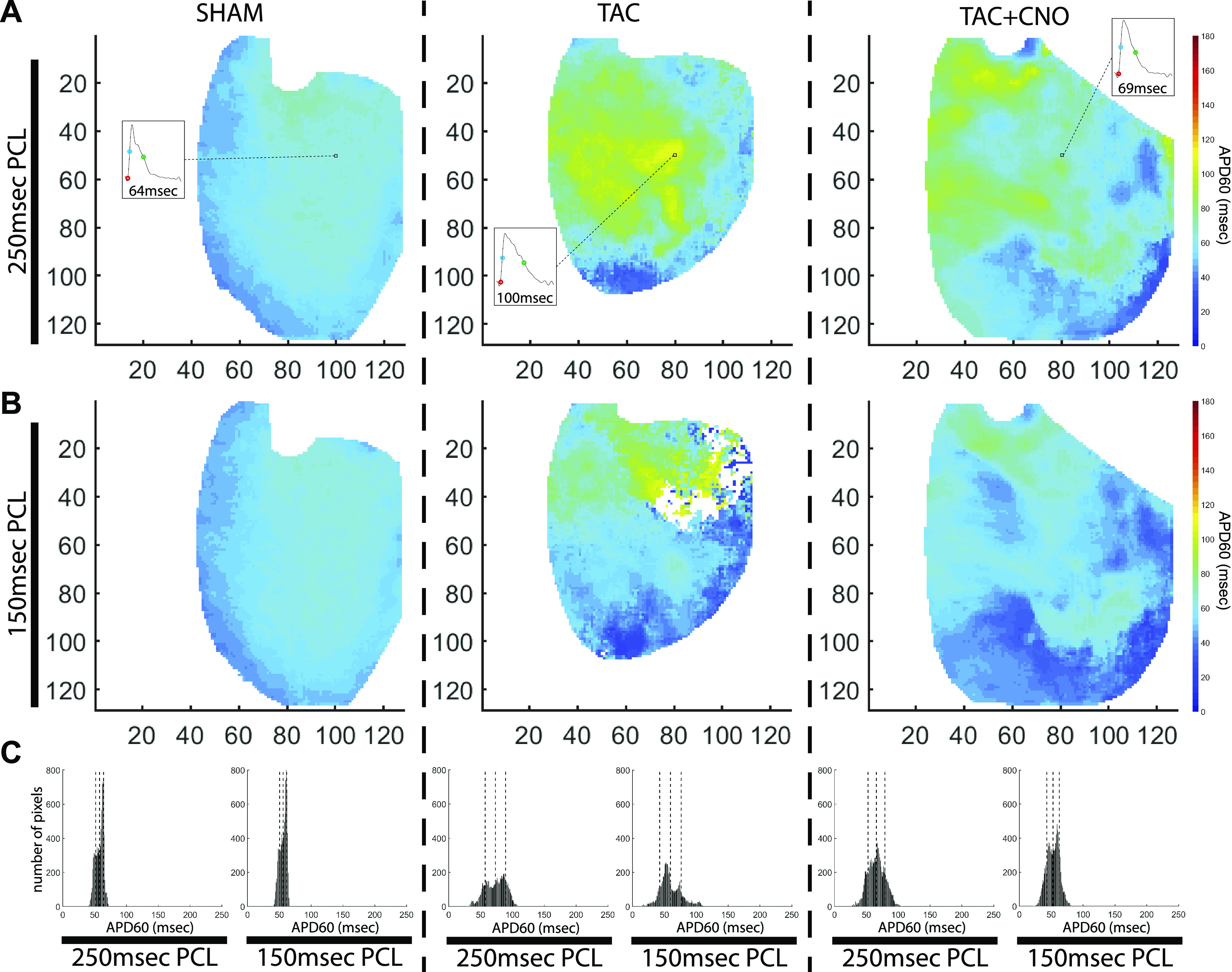
Heterogeneous APD_60_ maps. *A*: per-pixel APD_60_ mean maps with insets showing the last optical AP of the 250 ms PCL. In the insets, circles mark onset of the AP upstroke (red), AP activation (blue), and 60% of AP repolarization (green). Axes units are pixels, with 118 µm/pixel. *B*: per-pixel APD_60_ mean maps at 150 ms PCL. *C*: histograms of APD_60_ maps shown in *A* and *B*. Global mean and standard deviation are marked with dotted lines in each histogram. APD_60_, action potential duration at 60% repolarization; AP, action potential; PCL, pacing cycle length.

**Fig. 3.**
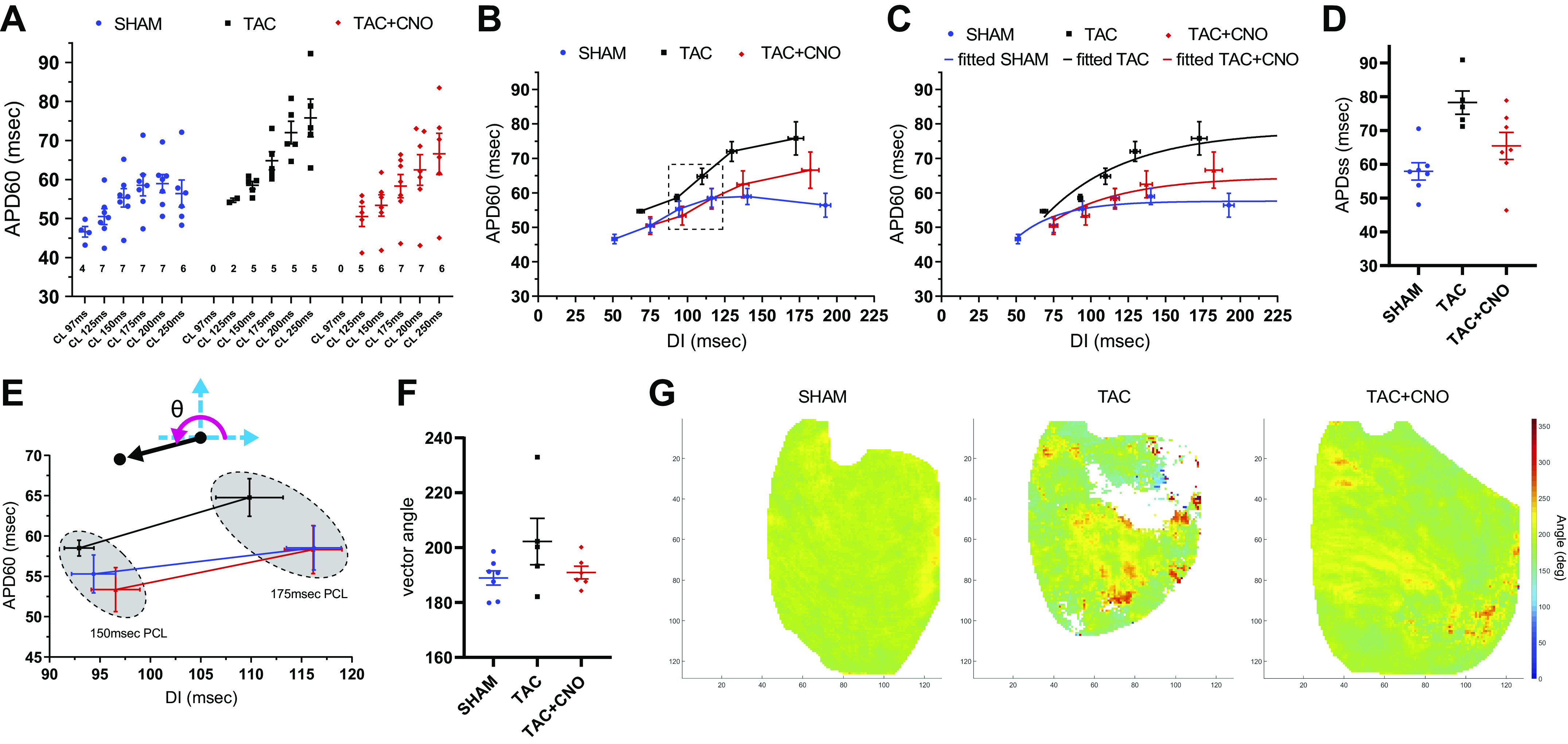
APD_60_ restitution analysis. *A*: global APD_60_ mean values for each PCL, with sample size listed below datapoints. *B*: APD_60_ restitution plot for captured PCLs using global APD_60_ (seen in *A*) and DI mean values. The dotted rectangle denotes the window of data shown in *E*. *C*: fitted APD_60_ restitution curves plotted with restitution points for each PCL. *D*: APD_ss_ computed from each heart’s fitted APD_60_ restitution curve. *P* values for group comparisons are listed in Table 1. *E*: restitution points for 175 ms and 150 ms PCL expanded from the dotted rectangle region shown in *B*. The definition of restitution vector angle (θ) is shown in the diagram above. *F*: APD_60_ restitution vector angles computed from 175 ms PCL to 150 ms PCL (Sham *n* = 7, TAC *n* = 5, and TAC + CNO *n* = 6). *P* values for group comparisons are listed in Table 1. *G*: representative APD_60_ restitution vector angle maps for each group. Axes units are pixels, with 118 µm/pixel. All error bars indicate standard error of the mean (SEM). APD_60_, action potential duration at 60% repolarization; APD_ss_, APD_60_ steady state; CNO, clozapine N-oxide; DI, diastolic interval; PCL, pacing cycle length; TAC, transverse-ascending aortic constriction.

TAC hearts had significantly increased global APD_60_ mean at 250 ms PCL compared with Sham hearts (*P* = 0.0108), but not when compared with TAC + CNO hearts (*P* = 0.1842). At 250 ms PCL, both TAC and TAC + CNO had heterogeneous long and short APD_60_ across the LV, as seen in per-pixel APD_60_ mean maps (Fig. 2*A* and Supplemental Fig. S1) and histograms (Fig. 2*C* and Supplemental Fig. S2). At the shorter 150 ms PCL, global APD_60_ mean converged for all groups (Fig. 3*A*); however, TAC and TAC + CNO hearts continued to exhibit APD_60_ heterogeneity across the LV (Fig. 2*B*). Per-pixel APD_60_ mean maps at 150 ms PCL in five of six TAC + CNO hearts demonstrated broader, more contiguous regions of lower APD_60_, compared with TAC hearts (Fig. 2*B* and Supplemental Fig. S1). The APD_60_ restitution curve for Sham was flatter and lower than the curves of TAC and TAC + CNO (Fig. 3, *A* and *B*). However, APD_ss_ from fitted APD_60_ restitution curves was significantly higher for TAC than Sham (*P* = 0.0010) and TAC + CNO (*P* = 0.0220) (Fig. 3, *C* and *D*). The transition from 175 ms to 150 ms PCL of the APD_60_ restitution curve was quantified by restitution vector angle (Fig. 3*E*). Although not significant, TAC hearts had higher angles than Sham (*P* = 0.0619) and TAC + CNO (*P* = 0.1176) hearts (Fig. 3*F*). Regions of increased vector angle are observed in at least four of five TAC hearts (Fig. 3*G* and Supplemental Fig. S3).

#### Conduction velocity.

**Fig. 4.**
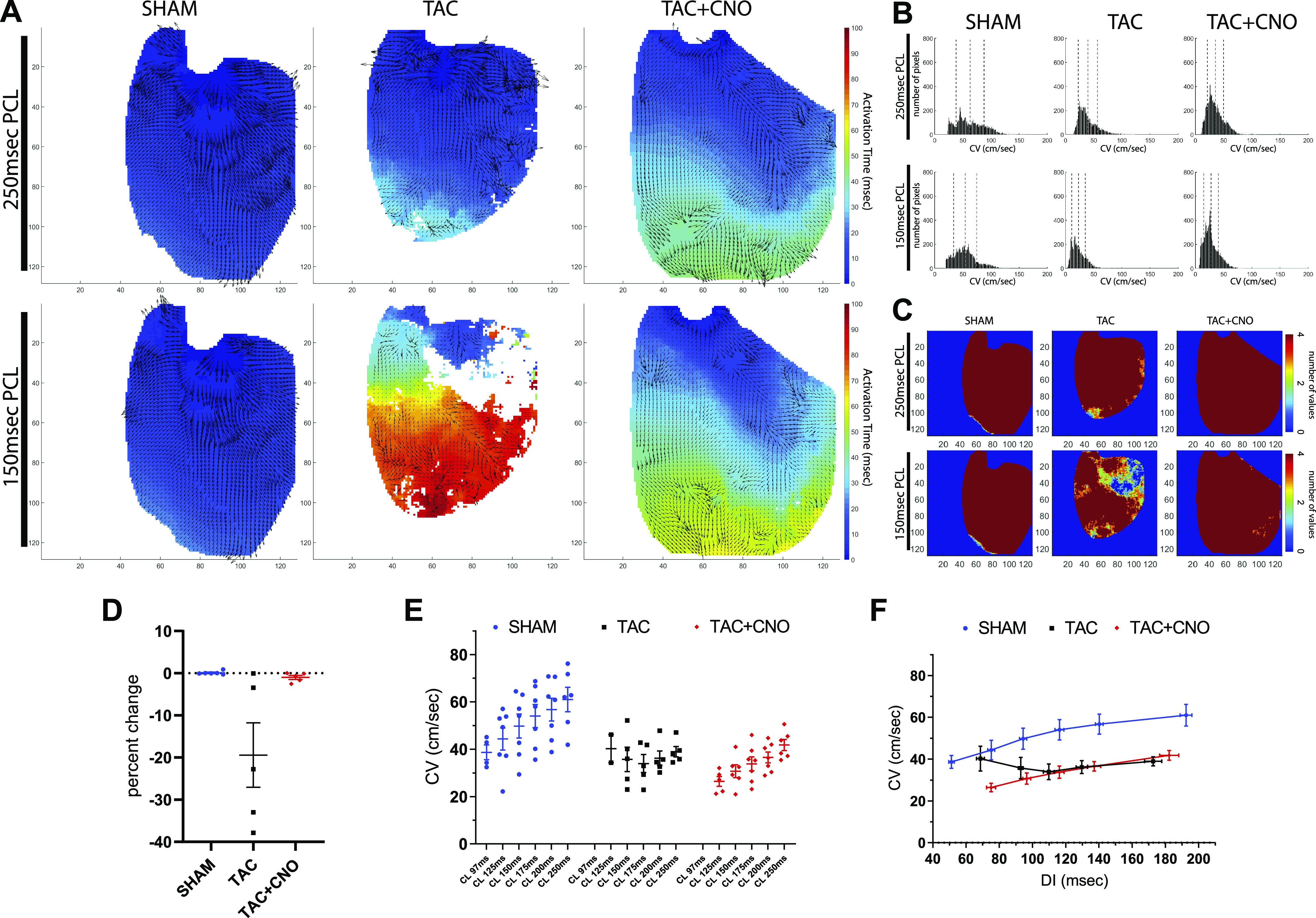
Disrupted conduction continuity. *A*: AT map with overlaid CV vectors for the last AP of 250 ms and 150 ms PCLs. Axes units are pixels, with 118 µm/pixel. *B*: CV histograms for the per-pixel means of the last four beats of 250 ms and 150 ms PCLs for the hearts shown in *A*. Global mean and standard deviation are marked with dotted lines in each histogram. *C*: conduction loss maps representing the number of values used at each per-pixel APD_60_ mean for both 250 ms and 150 ms PCLs. Axes units are pixels, with 118 µm/pixel. *D*: percent change between 250 ms and 150 ms in the number of pixels with four values used for per-pixel APD_60_ mean. Sham, *n* = 6; TAC, *n* = 5; and TAC + CNO, *n* = 5. *E*: CV global mean values for each captured PCL. *F*: CV restitution plot for captured PCLs using global CV (seen in *E*) and DI mean values. Sample size for *E* and *F* same as Fig. 3*A*. All error bars indicate standard error of the mean (SEM). APD_60_, action potential duration at 60% repolarization; AP, action potential; AT, activation time; CNO, clozapine-*N*-oxide; CV, conduction velocity; PCL, pacing cycle length.

Global mean CV in TAC hearts was significantly lower (*P* = 0.0010) than Sham hearts at 250 ms PCL, and TAC hearts demonstrated complex activation wave propagation compared with Sham hearts (Fig. 4, *A* and *B*, and Supplemental Fig. S4). Additionally, CV restitution values for TAC and TAC + CNO were consistently lower than that of Sham across PCLs (Fig. 4, *E* and *F*). In TAC hearts, a reduction in PCL from 250 ms to 150 ms resulted in fewer per-pixel AP detections across 4 beats, which demonstrates increased conduction loss compared with Sham and TAC + CNO hearts (Fig. 4*C* and Supplemental Fig. S1). Across TAC hearts, this increased conduction loss varied significantly, compared with Sham and TAC + CNO hearts, as demonstrated by the Brown–Forsythe equal variance test (*P* = 0.0012) (Fig. 4*D*). Additionally, at 125 ms PCL, 40% of TAC hearts captured pacing compared with 71% of TAC + CNO hearts and 100% of Sham hearts.

#### APD_60_ vs. DI dispersion.

**Fig. 5.**
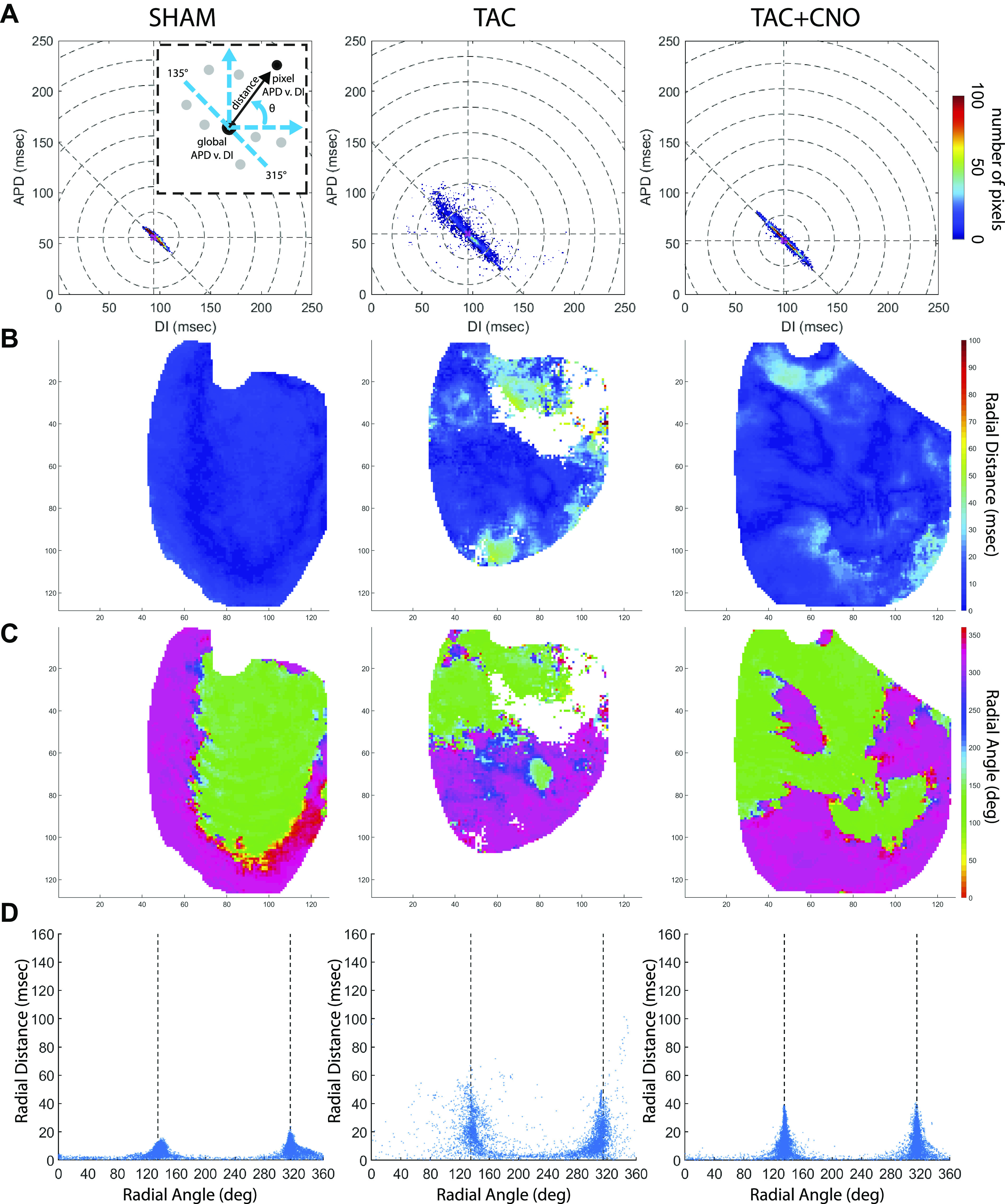
APD_60_ vs. DI dispersion analysis. *A*: APD_60_ vs. DI dispersion two-dimensional histograms for 150 ms PCL using each pixel’s per-pixel APD_60_ and DI mean value. Concentric dotted circles represent 25 ms radial distance from global mean restitution point. Dotted diagonal line represents the predicted PCL = APD + DI line. Schematic *inset* refreshes Fig. 1*C* concepts of radial distance (black arrow) and radial angle (θ). *B*: radial distance maps at 150 ms PCL. *C*: radial angle maps for 150 ms PCL. Axes units are pixels, with 118 µm/pixel. *D*: radial distance vs. radial angle scatter plots for 150 ms PCL with line marked for PCL = APD + DI. APD_60_, action potential duration at 60% repolarization; DI, diastolic interval; PCL, pacing cycle length.

**Fig. 6.**
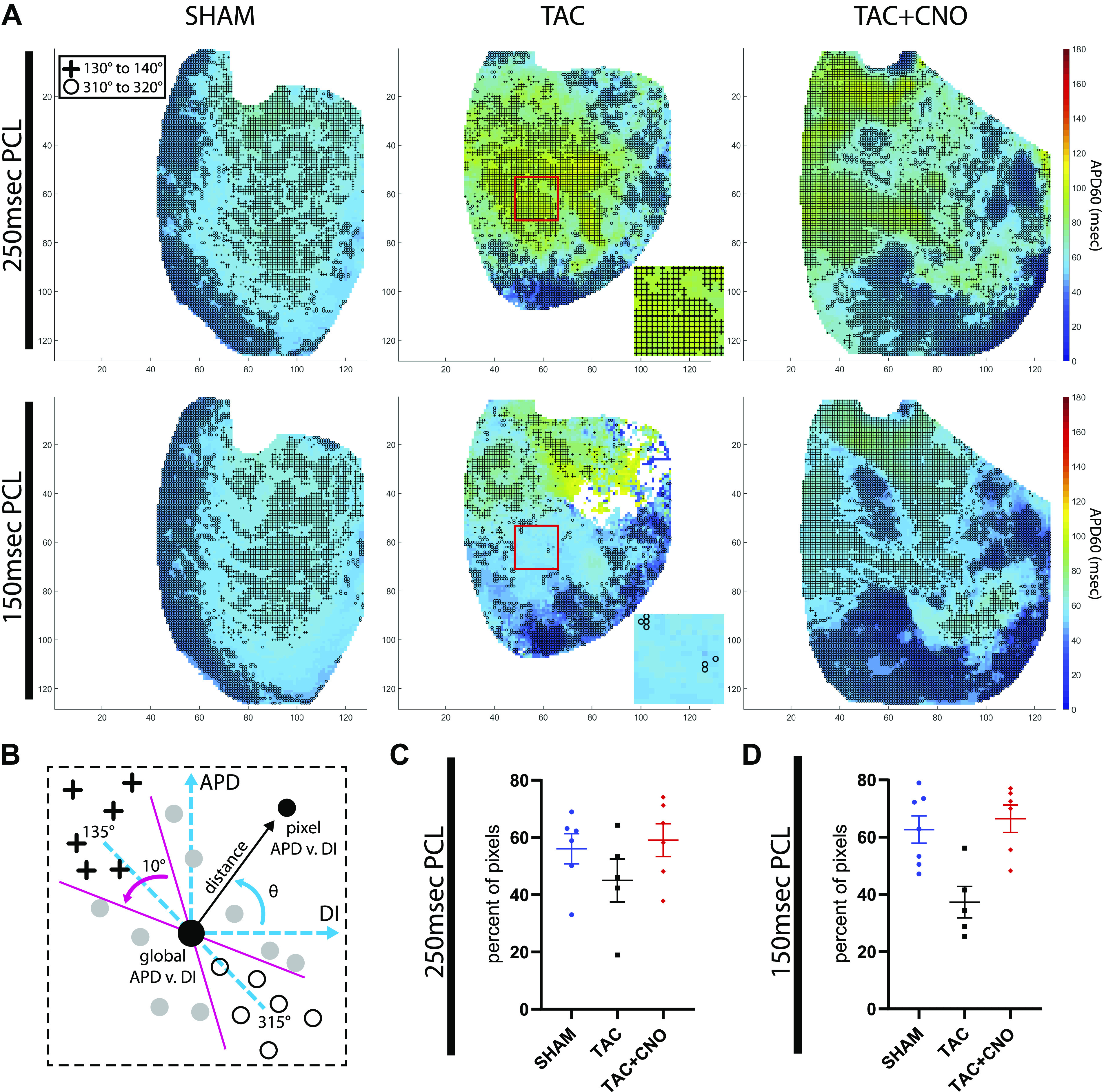
Segment analysis for rate responsive tissue. *A*: per-pixel APD_60_ mean maps for PCLs 250 ms and 150 ms with markers for pixels that are within the APD_60_ vs. DI 10° segments centered at 135° (crosses) and 315° (circles) along the predicted PCL = APD + DI line. Red box and corresponding *inset* for TAC highlights a region of increased APD_60_ vs. DI dispersion due to loss of pixels in 10° segments at 150 ms PCL. Axes units are pixels, with 118 µm/pixel. *B*: schematic of segment markers refreshing Fig. 1*C*. *C* and *D*: percent of total pixels lying within these 10° segments of the APD_60_ vs. DI dispersion for PCLs 250 and 150 ms. *P* values for group comparisons are listed in Table 1. Sample size for (*C*) Sham, *n* = 6; TAC, *n* = 5; and TAC + CNO, *n* = 6. Sample size for (*D*) Sham, *n* = 7; TAC, *n* = 5; and TAC + CNO, *n* = 6. All error bars indicate standard error of the mean (SEM). APD_60_, action potential duration at 60% repolarization; CNO, clozapine-*N*-oxide; DI, diastolic interval; PCL, pacing cycle length; TAC, transverse-ascending aortic constriction.

Each pixel’s repolarization rate response to a single PCL was quantified using APD_60_ versus DI dispersion analysis (Fig. 1*C*). At 150 ms PCL, TAC hearts exhibited greater dispersion (scatter) in the APD_60_ versus DI point cloud along the predicted PCL = APD + DI line compared with Sham and TAC + CNO hearts (Fig. 5*A* and Supplemental Fig. S5). APD_60_ versus DI radial distance and radial angle mapping amplified regional differential APD_60_ from the PCL’s global mean restitution point (Fig. 5, *B* and *C*, and Supplemental Fig. S5). Distance versus angle plots showed how pixels fell into 10° segments centered at 135° and 315° along the predicted PCL = APD + DI line (Figs. 5*D*, 1*C*, and 6*B*). Pixels inside these 10° segments identified epicardial regions that appropriately adapted to a PCL (Fig. 6*A*). At 250 ms PCL, TAC hearts did not significantly differ in percentage of pixels within these segments, compared with Sham and TAC + CNO hearts (Fig. 6*C*). However, at 150 ms PCL, TAC hearts had a significantly lower percentage of pixels within these 10° segments, compared with Sham (*P* = 0.0029) and TAC + CNO (*P* = 0.0013) hearts (Fig. 6*D*). In at least three of five TAC hearts, pixels in these 10° segments at 250 ms PCL subsequently fell outside the segments at 150 ms PCL (Fig. 6*A* *inset* and Supplemental Fig. S5).

## DISCUSSION

In this study, we measured LV epicardial electrophysiological parameters after 16 wk of pressure overload-induced heart failure in rats. We found that TAC elevated the APD_60_ restitution curve, increased APD_60_ versus DI dispersion, slowed conduction, and increased variance of epicardial conduction loss during dynamic pacing. We sought to blunt such TAC-induced electrophysiological changes by stimulating myocardial cholinergic activity via chronic activation of PVN oxytocin neurons. Our results show that cholinergic stimulation blunted TAC-induced electrophysiological alterations by reducing APD_60_ versus DI dispersion and the variance of epicardial conduction loss during dynamic pacing. Additionally, cholinergic stimulation reduced APD_60_ elevation in large epicardial regions as well as reduced fitted APD_ss_ compared with TAC.

### 

#### Prolonged action potential duration.

In this study, progressively increasing rate every 30 pulses revealed altered repolarization within the LV epicardium of TAC hearts. At 250 ms PCL, we observed a significant APD_60_ increase in TAC LV epicardial tissue compared with Sham (Fig. 3). This finding is consistent with many forms of heart failure at low heart rates (6). When reducing PCL to 150 ms, global APD_60_ mean of TAC hearts converged with that of Sham hearts. At yet shorter PCLs, observed conduction failure may be explained by the restitution slope hypothesis (45) where predicted increased restitution steepness due to elevated TAC APD_ss_, compared with Sham and TAC + CNO, increased the risk of conduction failure (28, 50).

Increased APD has been reported for ventricular myocytes isolated from failing hearts of multiple species (40, 47, 48), including humans (7). The primary cause of the delayed repolarization is a downregulation of repolarizing K^+^ currents (30, 37, 51, 54), which include the transient outward current (Ito) (12, 40, 43), the inward rectifier K^+^ current (IK1) (12, 40), and the delayed rectifier K^+^ current (IKr) (12, 36). Acute reduction of K^+^ current density is suggested to be caused by increased α_1_ adrenergic activation (1, 49) resulting from increased catecholamine levels during heart failure, with chronic current reductions additionally influenced by reduced K^+^ channel expression (12, 25). Reduced K^+^ current density combined with dysregulation of intracellular Ca^2+^ that occurs during heart failure (6) could also contribute to delayed repolarization in failing myocytes. In this scenario, the combination of reduced sarcoplasmic reticulum Ca^2+^ ATPase (SERCA) activity (20) and increased expression of the Na^+^/Ca^2+^ exchanger (NCX) (40, 53) that both occur during heart failure would favor increased inward NCX current that would balance outward K^+^ currents to delay repolarization and increase APD.

#### Cholinergic stimulation.

Mechanistically, increased cholinergic stimulation may inhibit TAC-induced increases in cardiac catecholaminergic activation. Our previous studies have shown diminished cardiac vagal neuron activity within the brainstem of rats with TAC-induced LV hypertrophy (10) and that chronic chemogenetic activation of PVN oxytocin neurons restores that activity (14). Upon increased cholinergic stimulation and myocardial acetylcholine release, M_2_ muscarinic stimulation could blunt elevated β_2_ and α_1_ adrenergic signaling (32). Increased α_1_ adrenergic signaling acutely suppresses K^+^ current density in isolated cardiomyocytes (1), providing longer APD. Ventricular patch-clamp studies show that IK1 and Ito currents are reduced by α_1_ adrenergic receptor regulation of CaMKII and PKC, respectively (49). Increased activation of the cardiac parasympathetic network may inhibit these adrenergic-induced reductions in repolarizing K^+^ currents if applied during cardiac insult. Chronic activation of the cardiac parasympathetic network also reduces myocardial inflammation during TAC (14, 17), which would mitigate impaired SERCA activity associated with high levels of inflammatory cytokines (15, 52). The resulting improvement in SERCA activity would favor improved regulation of intracellular Ca^2+^, reduced NCX expression, and reduced APD. These mechanisms may explain the reduction in APD_ss_ for TAC + CNO compared with TAC, which provides evidence of increased repolarizing K^+^ currents and decreased inward currents that together improved APD adaptation to increased heart rate. Further, supported by a trend of increased restitution vector angle in TAC compared with TAC + CNO, we expect curve steepness to significantly increase in TAC compared with TAC + CNO hearts if capture would have been maintained, consistent with studies of structural heart disease in humans (27) and in animal studies of steady-state LV APD analysis (45).

#### Regional repolarization.

We observed heterogeneous repolarization in the LV epicardium of both TAC and TAC + CNO hearts, as demonstrated in APD_60_ mean maps (Fig. 2 and Supplemental Fig. S1). However, in contrast to TAC hearts, regions with lower APD_60_ were larger in TAC + CNO hearts. These areas of lowered APD_60_ may suggest that cholinergic-induced relief of electrophysiological derangement during TAC might be more pronounced in areas of high cholinergic activity (24).

#### Regional depolarization and conduction.

In this study, 16 wk of TAC altered epicardial conduction, demonstrated by reduced conduction velocity in both TAC and TAC + CNO hearts across the PCLs. However, conduction loss variability in TAC hearts increased compared with TAC + CNO hearts (Fig. 4 and Supplemental Fig. S1). Cholinergic stimulation may maintain continuity of conduction during cardiac disease by blunting inflammation and collagen deposition that generate disruptive bundles of fibrosis (14, 17, 29).

#### APD_60_ vs. DI dispersion.

When analyzing APD_60_ versus DI dispersion, the predicted line of PCL = APD + DI provided a measure to quantify APD_60_ rate adaptation within a single PCL (Fig. 5). For example, this line predicts where a site of epicardium with shortened APD_60_ should fall, since it should correspond to a longer DI for a fixed PCL. Compared with TAC, cholinergic stimulation reduced deviation from this line at 150 ms PCL. A pixel would fall off the predicted line, for example, by having an APD and DI that together (APD + DI) differ from the PCL. This could be caused by local CV alterations that may occur in areas bordering conduction delays, where slight shifts in propagation direction would create localized variations in cycle lengths. APD responds to these local cycle lengths, establishing a new APD + DI relationship that is parallel and offset to the predicted PCL line. The location of the APD versus DI point along that new parallel line is dictated by the local tissue restitution properties. Increased incidence of offset from the predicted PCL line indicates decreased homogeneity of rate adaptation to the PCL, as we observed for TAC hearts. Additionally, a localized failure of 1:1 capture could drag a pixel off the predicted line and may explain how some pixels exceeded the maximum PCL window, as also observed for TAC hearts.

Importantly, fewer pixels falling within 10° segments surrounding the PCL = APD + DI line marked increased maladaptive epicardium upon increased rate in TAC hearts compared with TAC + CNO hearts (Fig. 6). Noise in the repolarization phase of the detected AP, particularly in APs with smaller amplitudes, may drag a pixel off the PCL = APD + DI line. Differential RH237 staining and emission observed in the TAC and TAC + CNO hearts may increase this noise to provide artifactual large and small APD_60_. However, the lack of a consistent and clear correlation in plots of per-pixel APD_60_ mean versus per-pixel steady-state fluorescence intensity and variance in Sham, TAC, and TAC + CNO hearts (Supplemental Fig. S6) suggests that artifactual APD_60_ did not dictate the outcomes of this study due to appropriate noise exclusion.

#### Conclusions.

LV epicardial conduction, APD_60_, and APD_60_ versus DI dispersion during sequential reductions in PCL were significantly altered after 16 wk of TAC compared with healthy Sham controls. Chronic chemogenetic activation of hypothalamic oxytocin neurons for downstream cardiac cholinergic neuron stimulation improved electrophysiological adaptation to increases in pacing rate during the development of TAC-induced heart failure. In addition to reducing APD_ss_, activation of cardiac cholinergic neurons blunted APD_60_ elevation in large epicardial regions, reduced variable conduction loss, and reduced APD_60_ versus DI dispersion during rapid pacing. These results provide insight into the electrophysiological benefits of cholinergic stimulation as a potential treatment for patients with heart failure.

## GRANTS

This work was supported by American Heart Association Predoctoral Fellowship 18PRE34030376 (to F. M. Zasadny), American Autonomic Society Postdoctoral Fellowship (to J. Dyavanapalli), and National Heart, Lung, and Blood Institute Grants R01 HL133862 and R01 HL146169 (to M. W. Kay. and D. Mendelowitz).

## DISCLOSURES

No conflicts of interest, financial or otherwise, are declared by the authors.

## AUTHOR CONTRIBUTIONS

F.M.Z., J.D., D.M., and M.W.K. conceived and designed research; F.M.Z. and J.D. performed experiments; F.M.Z. analyzed data; F.M.Z. and M.W.K. interpreted results of experiments; F.M.Z. prepared figures; F.M.Z. drafted manuscript; F.M.Z., J.D., N.M.D., D.M., and M.W.K. edited and revised manuscript; F.M.Z., J.D., N.M.D., D.M., and M.W.K. approved final version of manuscript.
